# Exogenous Fecal Microbiota Transplantation from Local Adult Pigs to Crossbred Newborn Piglets

**DOI:** 10.3389/fmicb.2017.02663

**Published:** 2018-01-09

**Authors:** Luansha Hu, Shijie Geng, Yuan Li, Saisai Cheng, Xiongfeng Fu, Xiaojing Yue, Xinyan Han

**Affiliations:** Key Laboratory of Animal Nutrition and Feed Science in East China, Ministry of Agriculture, College of Animal Science, Zhejiang University, Hangzhou, China

**Keywords:** exogenous fecal microbiota transplantation, gut microbiota, intestinal morphology, intestinal barrier function, newborn piglets

## Abstract

This study was conducted to investigate the effect of exogenous fecal microbiota transplantation on gut bacterial community structure, gut barrier and growth performance in recipient piglets. Twelve litters of Duroc × Landrace × Yorkshire piglets of the same birth and parity were weighed and divided into two groups. One group (recipient piglets) was inoculated orally with fecal microbiota suspension of healthy adult Jinhua pigs daily from day 1 to day 11. The other (control) was given orally the same volume of sterile physiological saline at the same time. The experiment lasted 27 days. The results showed that the relative abundance of *Firmicutes, Prevotellaceae, Lachnospiraceae, Ruminococcus, Prevotella*, and *Oscillospira* in the colon of recipient piglets was increased. *Proteobacteria, Fusobacteriaceae, Clostridiaceae, Pasteuriaceae, Alcaligenaceae, Bacteroidaceae, Veillonellaceae, Sutterella, Escherichia*, and *Bacteroides* in the colon of recipient piglets were decreased. An average daily weight gain of recipient piglets was increased, and diarrhea incidence of the recipient was decreased during the trial. Intestinal morphology and tight junction barrier of recipient piglets were improved. The optical density of sIgA^+^ cells, the number of goblet cells and relative expressions of MUC2 in the intestinal mucosa of recipient piglets were enhanced. Protein expressions of β-defensin 2 and mRNA expressions of TLR2 and TLR4 in the intestinal mucosa of recipient piglets were also increased. These findings supported that the exogenous fecal microbiota had significant effects on animal’s growth performance, intestinal barrier function, and innate immune via modulating the composition of the gut microbiota.

## Introduction

Intestinal microbial flora plays an important role in human and animal health, it has attracted more and more attention in recent years. Gut contains a tremendous amount of microorganisms, which makes a very big contribution to individual health. A healthy human intestine contains more than 10^14^ bacteria, which is 10 times the number of all cells in the body (Koboziev et al., 2014). As it has a close relationship with absorption of nutrients, colonization resistance, development of the immune system and other functions in host, intestinal microbial flora is known as an essential organ (Moya and Ferrer, 2016). Although gut microbiota resides in the intestine, it can cause systemic effects (Ho et al., 2015). Not only the intestine got affected by the change of gut microbiota diversity and balance, but the whole body system could get changed (Ho et al., 2015). Diseases caused by imbalance of intestinal microbiota can be treated by treatments involving microbiota, such as probiotics, prebiotics, synbiotics, and FMT (fecal microbiota transplantation) (Ho et al., 2015). FMT refers to the transplantation of fecal suspension from healthy individuals into the gastrointestinal tract of the patient, in order to achieve the treatment of gastrointestinal diseases. The first record about FMT was a Chinese physician named Ge Hong who used FMT to treat food poisoning and severe diarrhea in Jin Dynasty, so far, the medical profession has never stopped exploration and application of FMT technology. The first randomized controlled trial of FMT was reported in 2012, in contrast to vancomycin, infusion of donor fecal bacteria was significantly more effective in treating refractory recurrent *Clostridium difficile* infection (van Nood et al., 2013). In addition, it also became a tool for functional “knock-in” studies of the microbiota in animal models (Bojanova and Bordenstein, 2016). However, the connections between FMT, the structural changes of gut microbiota and improvements of intestinal health in recipients as well as the mechanism underlying these effects are not fully illustrated.

Intestinal microbiota of a newborn is in an unstable state, its diversity increase gradually and is vulnerable to external environment and other factors (Clemente et al., 2012; Pabst et al., 2016). Therefore, newborn piglets are usually used as a model, which can be a good animal model for studying human nutrition and physiology (Puiman and Stoll, 2008; Merrifield et al., 2011). However, pig diarrhea can cause sucking piglets to die, which would bring significant economic losses to the pig farm. Jinhua pig, one of the most well-known local breeds in China, has the advantages of good meat quality, high rate of reproduction and a better ability to resist the challenge of enterotoxigenic *Escherichia coli* (ETEC) K88 and maintain the intestinal physiology homeostasis (Gao et al., 2013). At the present study, newborn piglets were selected as the model, to explore the effect of exogenous fecal microbiota on animal health from the perspective of gut microbiota, intestinal physiological function and growth performance. We hypothesized that exogenous fecal microbiota transplantation would modulate the structure of the gut microbiota, improve intestinal barrier and immune function in newborn piglets. Therefore, this study might provide novel insights in understanding this efficient therapy strategy for various gastrointestinal diseases.

## Materials and Methods

### Preparation of Fecal Microbiota Suspension of Donor Pigs

Jinhua pigs with no antibiotics treated within 3 months were used in this experiment as fecal donors. The detection of hog cholera virus, porcine circovirus-2, porcine reproductive, and respiratory syndrome virus, pseudorabies virus, foot and mouth disease virus, swine erysipelas, *Mycoplasma hyopneumoniae* in donor pigs were negative. The fecal suspension was prepared as described by Pang et al. (2007), which was quickly aspirated and transferred to sterile Eppendorf tubes and stored at -80°C.

### Transplantation of Fecal Microbiota Experiment and Animal Management

The present study followed the institutional and national guidelines for the care and use of animals. All animal management experimental procedures involving animal care and sampling were approved by the Animal Care and Use Committee of Zhejiang University. A total of twelve litters (9–10 piglets per litter) of DLY(Duroc × Landrace × Yorkshire) piglets of the same birth day and parity were weighed and divided into two groups from birth(each group had six pens with two pigs per pen). One group (recipient piglets) was inoculated orally with 1.5 ml fecal suspension daily from day 1 to day 11. The other (control) was given orally the same volume of sterile physiological saline at the same time. All piglets were breast-fed by sows and weaned at day 28. All of the piglets were inoculated orally using a syringe attached to a polyethylene tube at 6 am in the morning. The two groups were kept in two separate pig houses. And the conditions (temperature, humidity and other conditions) of the two pig houses were controlled consistently. The experiment lasted 27 days. Six piglets were randomly selected from each group and euthanized by sodium pentobarbital (50 mg/kg body weight) at day 12 and day 27. Piglets were weighed individually at the start and end of the experiment, and average daily gain (ADG) was calculated for both groups. The number of piglets with diarrhea and its duration were observed and recorded during the experiment. Diarrhea was defined as liquid consistency over a minimum of 2 consecutive days. The incidence of diarrhea (%) was calculated as the total number of diarrheal piglets during the period divided by the total number of piglets multiplies duration of the trial.

### Collections of Samples

Intestinal samples and colon contents were collected when the piglets were euthanized. For intestinal morphological analysis, the duodenal (about 5 cm from the pyloric-duodenal junction), mid-jejunal and ileal (about 10 cm from the ileal-caecal junction) segments were sampled and fixed in buffered formalin (10%) at 4°C for morphometric analysis. Jejunum tissues were fixed overnight in a 2.5% glutaraldehyde solution at 4°C and then these samples were treated for observation by electron microscopy. The mucosa samples from the ileum and colon were harvested by scraping with a sterile glass microscope slide, rapidly frozen in liquid nitrogen and stored at -80°C for further analysis. Colon contents (from the middle of the colon) were collected from piglets. Digesta samples were placed into sterile polypropylene centrifuge tubes, snap frozen in liquid nitrogen and kept frozen at -80°C until DNA extraction.

### Extraction of DNA and 16S rRNA Amplicon Sequencing

Total DNA was extracted and purified from about 200 mg of individual colon contents using the QIAamp stool DNA Mini kit (QIAGEN, United States) according to the manufacturer’s instructions. Sequencing was performed at the Novogene Bioinformatics Technology Co. Ltd., Beijing, China. DNA was amplified by using the 515f/806r primer set (515f:5′-GTG CCA GCM GCC GCG GTA A-3′, 806r:5′-XXX XXX GGA CTA CHV GGG TWT CTA AT-3′). PCR reactions were carried out in 30 μL reactions with 15 μL of Phusion^®^ High-Fidelity PCR Master Mix (New England Biolabs); 0.2 μM of forward and reverse primers, and about 10 ng template DNA. Thermal cycling consisted of initial denaturation at 98°C for 1 min, followed by 30 cycles of denaturation at 98°C for 10 s, annealing at 50°C for 30 s, and elongation at 72°C for 30 s. Finally 72°C for 5 min. PCR products were purified by using the QIAquick Gel Extraction Kit (QIAGEN, Dusseldorf, Germany). Sequencing libraries were generated using NEB Next^®^ Ultra^TM^ DNA Library Prep Kit for Illumina (NEB, United States) following manufacturer’s recommendations and index codes were added. The library quality was assessed on the Qubit@ 2.0 Fluorometer (Thermo Scientific) and Agilent Bioanalyzer 2100 system. At last, the library was sequenced on an Illumina HiSeq platform and 250 bp paired-end reads were generated.

### Examination of Intestinal Morphological

The samples were embedded with paraffin wax and sectioned at 5 μm on a rotary microtome. Then, the sections were stained with hematoxylin and eosin. Villus height and crypt depth (V/C) were evaluated under a light microscope using a 1/100 ocular scale (Olympus, Japan). Morphological indices were determined using image processing and analysis system (Version 1, Leica Imaging Systems Ltd., Cambridge, United Kingdom) according to the technique of Han et al. (2014).

The mid-jejunum specimen fixed with 2.5% glutaraldehyde was washed with phosphate buffer three times, then fixed with 1% OsO_4_ solution in (pH 7.0) for 2 h and washed with phosphate buffer (0.01M) three times. After that, the specimens were dehydrated by a graded series of ethanol (30, 50, 70, 80, 90, and 95% respectively). The segments were transferred to the mixture of alcohol and iso-amyl acetate (v:v = 1:1) for 30 min, and then transferred to iso-amyl acetate (100%) for 1 h. After being dehydrated with liquid CO_2_ by a critical point dryer (Hitachi Model HCP-2, Japan), the segments were coated with gold-palladium and observed by Scanning Electron Microscope (SEM, Philips Model TM-1000, Japan).

### Relative Expression of mRNA by Real-time PCR

The mRNA levels of TLR2, TLR4, and MUC2 were determined by real-time PCR. Total RNA was extracted from ileum and colon mucosa using the TRIzol^®^ Plus RNA Purification Kit following the manufacturer’s guidelines. RNA was spectrophotometrically quantified and its integrity was verified by agarose gel electrophoresis. Reverse transcription using the SuperScript^TM^ III First-Strand Synthesis SuperMix for qRT-PCR was carried out following the manufacturer’s instructions. Quantitative analysis of PCR was carried out on a CFX384 real-time fluorescent quantitative PCR system using a Power SYBR^®^ Green PCR Master Mix, according to the manufacturer’s instructions. The primers used were presented in **Table 1**. Each sample was repeated three times and the relative expression level of each gene was analyzed statistically by 2^-ΔΔC_T_^ (Livak and Schmittgen, 2001).

**Table 1:** Real-time PCR primers and conditions.

Gene	Genbank accession	Primer sequences (5′ to 3′)	Size (bp)	Annealing (°C)
Pig Muc2	XM_013989745.1	ACCCCTGCTCCCTCAACATC	76	60
		GGGGTCCCCGTCTTCTTCAA		
Pig TLR2	NM_213761.1	GAGCACTTCCAGCCTCCCTT	118	60
		GCACGAAGATGGTTTTCTGGCTCTT		
Pig TLR4	NM_001113039.1	CTTGCAGTGGGTCAAGGACCA	133	60
		GACGGCCTCGCTTATCTGACAG		
Pig GAPDH	NM_001206359.1	CCAGGGCTGCTTTTAACTCTG	104	60
		GTGGGTGGAATCATACTGGAACAT		

### Relative Expression of Protein by Western Blot

The protein expressions of β-defensin 2, MUC2, tight junction proteins: ZO-1 and Occludin in the intestine were determined by western blot. Total protein extraction was performed using T-PER Tissue Protein Extraction Reagent (Thermo Pierce, 78510), protein quantification was then performed using the BCA Quantitation Kit. After the process of SDS-page electrophoresis analysis, transferred membranes, T-TBS (containing 5% non-fat dry milk or BSA) was added to the membrane and blocked at room temperature for 1 h. Antibody (1: 100) was added and incubated overnight at 4°C, then washing the membrane. Secondary antibody [Goat anti-Mouse IgG (H + L)] was added and incubated at room temperature for 1 h, and then washed the membrane. SuperSignal^®^ West Dura Extended Duration Substrate was used for Western blot detection. The optical density of the bands was analyzed using Image J software. The β-actin was used as an internal control, which exhibited no difference between the groups. The relative abundance of each target protein was expressed as the ratio of target protein/β-actin protein.

### Detection of Goblet Cells and sIgA^+^ Cells

The morphology and distribution of goblet cells in the intestinal epithelium were observed by light microscopy after the wax were stained with PAS according to the procedures of van Es et al. (2005). Moditec camera software was applied to take pictures. 5–10 complete intestine villi were selected from each tissue slice, and the number of goblet cells of per 100 intestinal epithelial cells was counted. Moreover, ileum and colon tissue sections were stained by immunohistochemical method according to the procedures of (Xiao et al., 2013). The average integrated optical density of sIgA^+^ cells in the ileum and colon mucosa was detected by using an image analysis system.

### Statistics

According to the Barcode sequence and the PCR amplification primer sequence, the sample data were separated from the down-machine data, and the Barcode and primer sequences were cut off using FLASH (V1.2.7)^1^. The original Tags data (Raw Tags) was obtained after the sample was spliced. The spliced Raw Tags, need to undergo a rigorous filtering to get high quality Tags (Clean Tags). Refer to Qiime (V1.7.0)^2^, the flow of Tags Quality Control, the procedure as follows: (a) acquirement of Tags: The Raw Quality was cut off at the first low mass base from the continuous low mass (the default quality threshold was ≤ 19) to the set length (default length value was 3). (b) The length filter of Tags: Further filtering out the value of which the continuous high quality base length is less than 75% of the length of the interceptive Tags. The sequences were clustered into OTUs (Operational Taxonomic Units) with 97% consistency, and a representative sequence of OTUs was selected. Species annotation of OTUs representative sequences was performed with RDP Classifier method and Green Gene database. The Alpha diversity analysis included Shannon index and Chao1. Data were analyzed using the SAS statistical package (SAS Institute, Cary, NC, United States). Heatmaps were visualized using the R software, log 10 -transformation was applied on the bacterial relative abundance data matrix. Data are expressed as the mean ± SEM, and all mean values were tested using Student’s *t*-test. A value of *P* < 0.05 was considered statistically significant.

## Results

### 16S rRNA Analysis of Bacterial Communities

**FIGURE 1:**
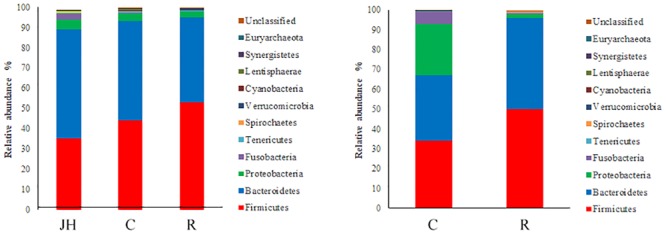
16S rRNA gene analysis revealed the colonic bacterial community structure of piglets orally treated with or without exogenous fecal microflora (relative abundance of microflora in the level of phylum.) JH, Jinhua pigs. C, Control piglets. R, Recipient piglets.

The information of OTU number and alpha diversity indexes of intestinal microbiota in piglets is presented in **Table 2**. There was no significant difference in OTU number, Shannon and Chao1 index between the recipient piglets and control piglets both on days 12 and 27 (*P* > 0.05). Relative abundance of gut microbial composition in the level of phylum was shown in **Figure 1**. The gut microbiotas of all pigs were dominated by *Firmicutes, Bacteroidetes, Proteobacteria*, and *Fusobacteria*. *Firmicutes* and *Bacteroides* accounted for 89–98% and were the absolute superiority in gut microbial flora composition of piglets. The results showed, compared with control piglets, *Firmicutes* of recipient piglets were increased (*P* < 0.05) and a decreasing trend of *Bacteroidetes* was found in recipient piglets at day 12 (*P* > 0.05). At day 27, recipient piglets had higher *Firmicutes* (*P* < 0.05) than control piglets. *Proteobacteria* of control piglets were significantly higher than that of recipient piglets (*P* < 0.05).

**Table 2:** The alpha diversity index of intestinal microflora in piglets.

Items	Day 12			Day 27	
	JH	Control piglets	Recipient piglets	Control piglets	Recipient piglets
OTU	843	824 ± 248	791 ± 226	1052 ± 334	1243 ± 378
Chao 1	1201	917 ± 128	897 ± 190	1214 ± 225	1342 ± 292
Shannon	6.6	7.2 ± 1.2	7.0 ± 0.8	7.3 ± 1.0	8.0 ± 1.8

**FIGURE 2:**
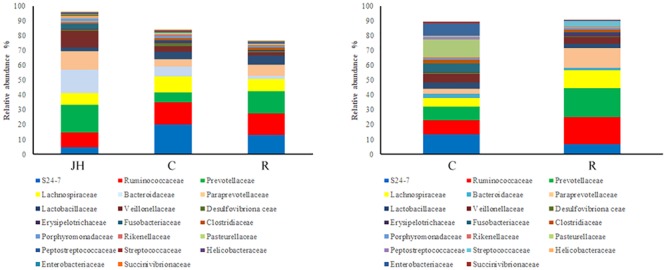
16S rRNA gene analysis revealed the colonic bacterial community structure of piglets orally treated with or without exogenous fecal microflora (relative abundance of microflora in the level of family.) JH, Jinhua pigs. C, Control piglets. R, Recipient piglets.

Relative abundance of gut microbial flora composition in the level of the family is shown in **Figure 2**. *Prevotellaceae* and *Paraprevotellaceae* were significantly increased of recipient piglets both on days 12 and 27 (*P* < 0.05). *Fusobacteriaceae, Clostridiaceae, Pasteuriaceae, Alcaligenaceae, Bacteroidaceae*, and *Veillonellaceae* were lower than the control on day 27 *(P <* 0.05*).* Compared with control piglets, there was an increasing trend of *Lactobacillaceae* of recipient piglets both on days 12 and 27 (*P* > 0.05).

**FIGURE 3:**
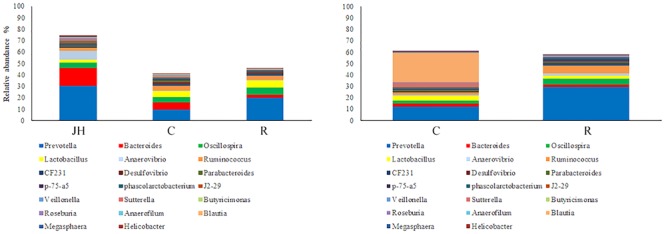
16S rRNA gene analysis revealed the colonic bacterial community structure of piglets orally treated with or without exogenous fecal microflora (relative abundance of microflora in the level of genus.) JH, Jinhua pigs. C, Control piglets. R, Recipient piglets.

**FIGURE 4:**
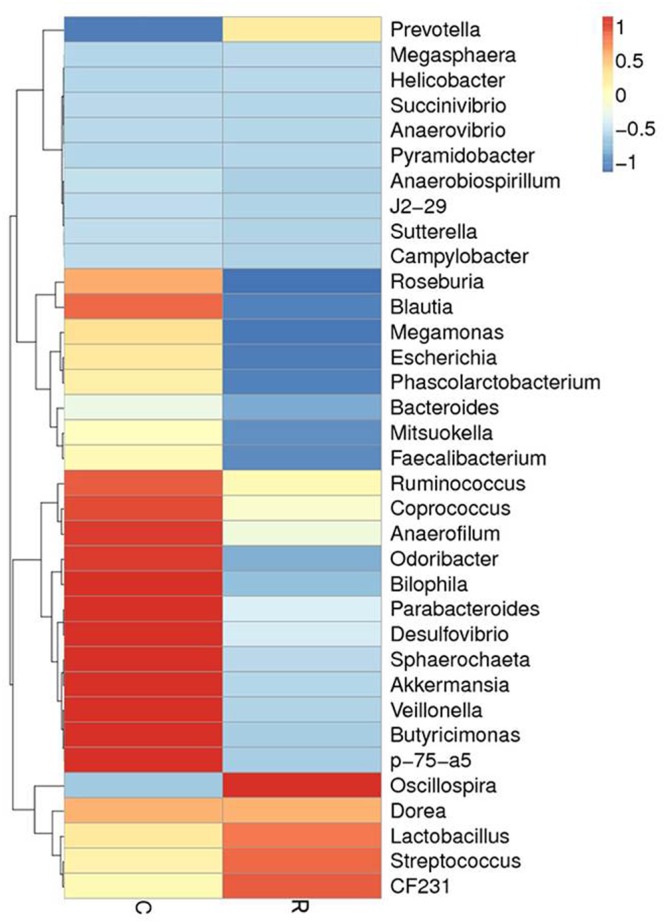
The distribution of luminal bacteria in the colonic digesta at day 12. Taxonomic profiles of the microbial communities of the colon were analyzed from genera. Pigs with the highest and lowest bacterial levels are red and green, respectively. C, Control piglets. R, Recipient piglets.

**FIGURE 5:**
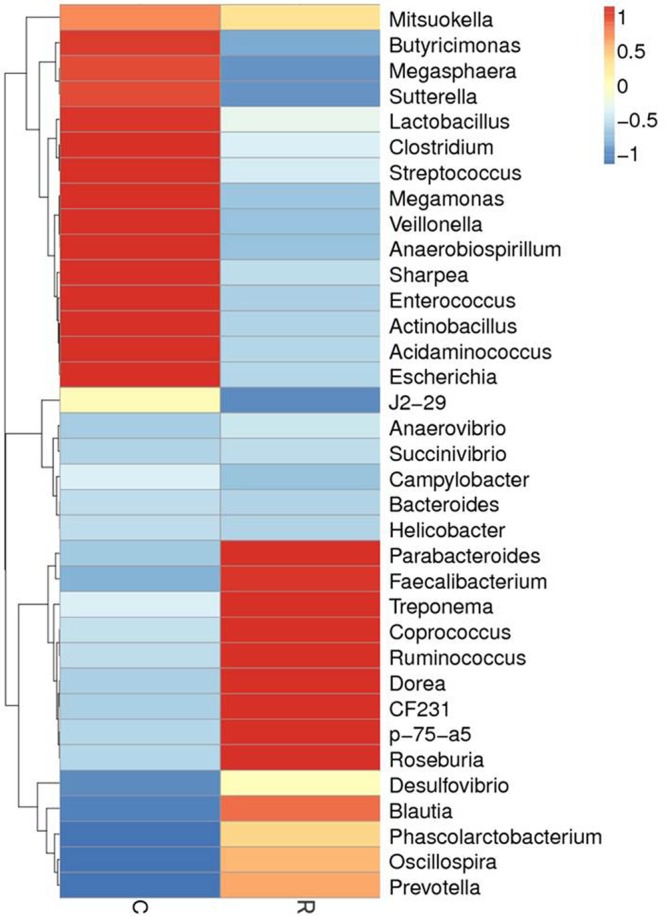
The distribution of luminal bacteria in the colonic digesta at day 27. Taxonomic profiles of the microbial communities of the colon were analyzed from genera. Pigs with the highest and lowest bacterial levels are red and green, respectively. C, Control piglets. R, Recipient piglets.

Relative abundance of gut microbial flora composition in the level of genus is shown in **Figure 3**. Taxonomic profiles of the microbial communities in the colon were analyzed from genera and the figures are shown in **Figures 4, 5**. Compared with control, *Prevotella, Oscillospira, CF231* were increased, *Bacteroides* were decreased of recipient piglets both on days 12 and 27 (*P* < 0.05). *Ruminococcus* were increased while *j2-29, Sutterella*, and *Escherichia* were decreased of the recipient piglets at day 27 (*P* < 0.05).

### Weight Gain and the Incidence of Diarrhea

**FIGURE 6:**
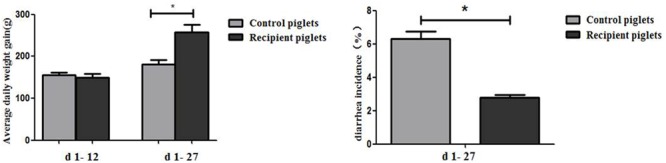
Weight gain and the incidence of diarrhea in piglets orally treated with or without exogenous fecal microflora. Data are expressed as the mean ± SEM. ^∗^*P* < 0.05.

Diarrhea and average daily weight gain of piglets are shown in **Figure 6**. At days 1–12, there were no significant differences of ADG between control and recipient piglets (*P* > 0.05). At days 1–27, ADG of the recipient piglets were higher than that of the control piglets (*P* < 0.05). Compared with control piglets, diarrhea incidence of recipient piglets was significantly decreased (*P* < 0.05).

### Intestinal Histology and Morphology

**FIGURE 7:**
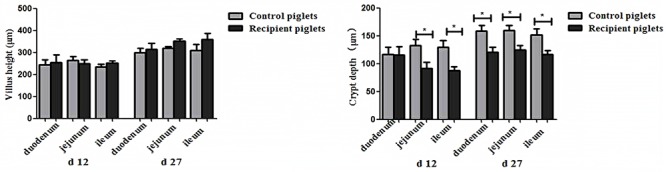
Duodenal, jejunal and ileal morphology of piglets orally treated with or without exogenous fecal microflora. Data are expressed as the mean ± SEM. ^∗^*P* < 0.05.

**FIGURE 8:**
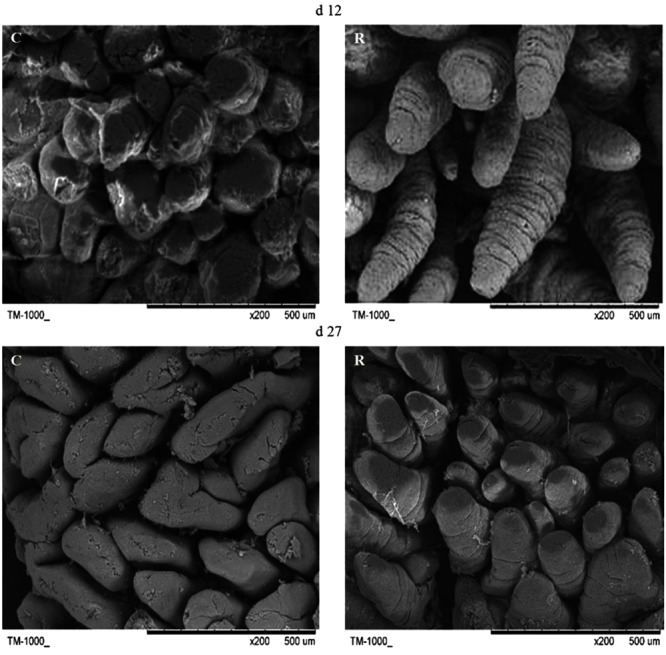
Scanning electron micrograph of jejunal mucosal surface of piglets orally treated with or without exogenous fecal microflora (200×, bar = 500 μm). C, Control piglets. R, Recipient piglets.

Small intestine villi height and crypt depth of piglets are shown in **Figure 7**. At days 12 and 27, the crypt depth of the recipient piglets was significantly decreased compared with control piglets (*P* < 0.05), while there was no significant difference in villus height of the small intestine between recipient and control piglets (*P* > 0.05). The morphology of villi in jejunum observed by scanning electron microscope was shown in **Figure 8**. Compared with control piglets, jejunum villus morphology of recipient piglets was arranged appropriately, the villi were long and finger-like, smooth and integrate.

### Relative Protein Expressions of Tight Junction Proteins in Intestine

**FIGURE 9:**
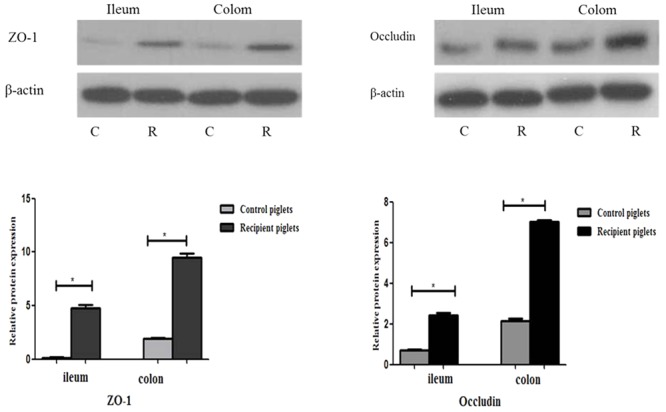
Relative protein expressions of tight junction proteins in ileum and colon mucosa of piglets orally treated with or without exogenous fecal microflora. Relative protein expressions were expressed as the ratio of the target protein and β-actin. C, Control piglets. R, Recipient piglets. Data are expressed as the mean ± SEM. ^∗^*P* < 0.05.

Relative protein expression of ZO-1 and Occludin in ileum and colon on days 12 and 27 is shown in **Figure 9**. Relative protein expression of ZO-1 and Occludin in ileum and colon of recipient piglets on days 12 and 27 were both higher than that of the control (*P* < 0.05).

### Number of Goblet Cells in Intestine

**FIGURE 10:**
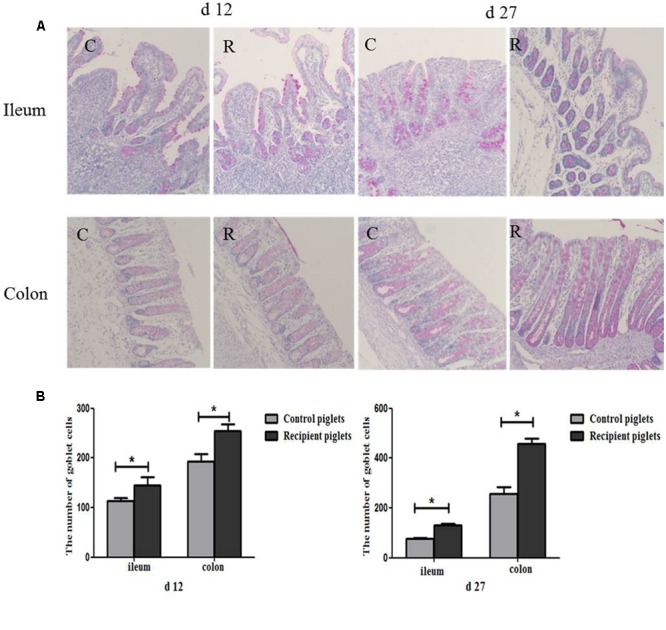
Distribution of goblet cells in the ileum and colon mucosa of piglets orally treated with or without exogenous fecal microflora. **(A)** The representative figure of goblet cells (PAS staining, 200×). **(B)** The number of goblet cells. C, Control piglets. R, Recipient piglets. Data are expressed as the mean ± SEM. ^∗^*P* < 0.05.

PAS staining of goblet cells is shown in **Figure 10**. Goblet cells distribution area in ileum and colon were broad and a large number of red secretory granules were observed both on days 12 and 27 of recipient piglets. The number of goblet cells in the ileum and colon was also shown in **Figure 10**. Compared with control piglets, the number of goblet cells in the ileum and colon of the recipient piglets was significantly increased both on days 12 and 27 (*P* < 0.05).

### Relative mRNA and Protein Expressions of MUC2 in Intestine

**FIGURE 11:**
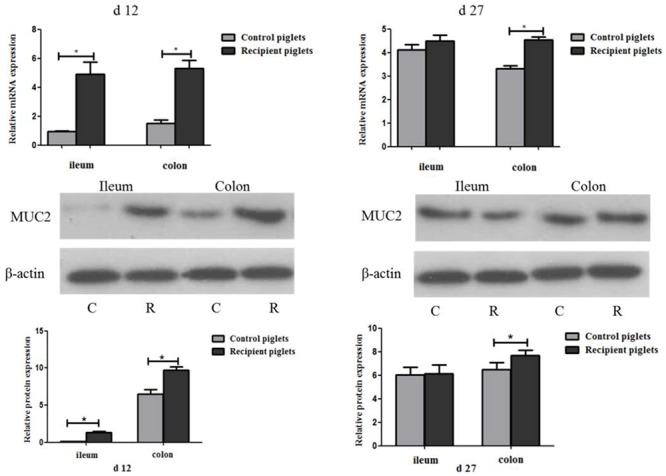
Relative mRNA and protein expressions of MUC2 in ileum and colon mucosa of piglets orally treated with or without exogenous fecal microflora. Relative protein expressions were expressed as the ratio of the target protein and β-actin. C, Control piglets. R, Recipient piglets. Data are expressed as the mean ± SEM. ^∗^*P* < 0.05.

The relative expression of mRNA and the protein of MUC2 are shown in **Figure 11**. At day 12, mRNA and protein expressions of MUC2 in the ileum and colon of the recipient piglets were both increased (*P* < 0.05). At day 27, mRNA and protein expressions of MUC2 in the colon of the recipient piglets were also increased (*P* < 0.05), though there was no significant change of it in the ileum (*P* > 0.05).

### Relative Protein Expressions of β-Defensin 2 in Intestine

**FIGURE 12:**
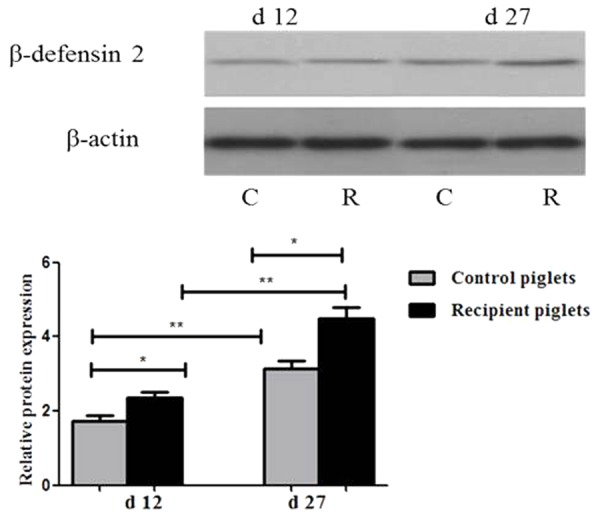
Relative protein expressions of β-defensin 2 in ileum mucosa of piglets orally treated with or without exogenous fecal microflora. Relative protein expressions were expressed as the ratio of the target protein and β-actin. C, Control piglets. R, Recipient piglets. Data are expressed as the mean ± SEM. ^∗^*P* < 0.05.

Relative protein expression of β-defensin 2 in the ileum mucosa on days 12 and 27 is shown in **Figure 12**. The expressions of β-defensin 2 in ileum of recipient piglets were higher than that of the control at days 12 and 27 (*P* < 0.05). The expressions of β-defensin 2 in ileum of piglets on day 27 were higher than that at day 12 both in control and recipient piglets (*P* < 0.05).

### The Concentration of sIgA in Intestine

**FIGURE 13:**
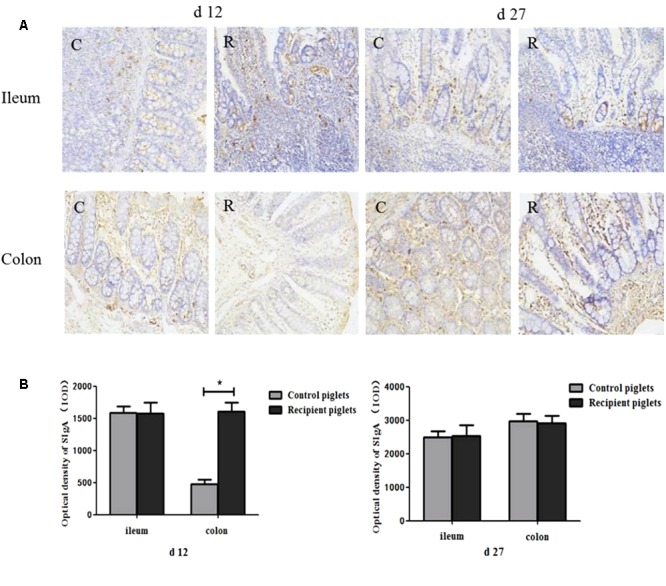
Concentration of sIgA in ileum and colon mucosa of piglets orally treated with or without exogenous fecal microflora. **(A)** sIgA^+^ cells immunohistochemical staining (immunohistochemical staining, 200×). **(B)** Integral optical density of sIgA^+^ cells. C, Control piglets. R, Recipient piglets. Data are expressed as the mean ± SEM. ^∗^*P* < 0.05.

The optical density of sIgA*^+^* cells in the intestinal mucosa is shown in **Figure 13**. There were positive staining both in ileum and colon of recipient piglets. At day 12, the optical density of sIgA*^+^* cells in the colon of the recipient piglets was increased (*P* < 0.05). There were no significant differences in the optical density of sIgA^+^ cells in the ileum (*P* > 0.05). At day 27, the optical density of sIgA^+^ cells in the ileum and colon showed no significant change of the recipient compared to control piglets (*P* > 0.05).

### Relative mRNA Expression of TLR2 and TLR4 in Intestine

**FIGURE 14:**
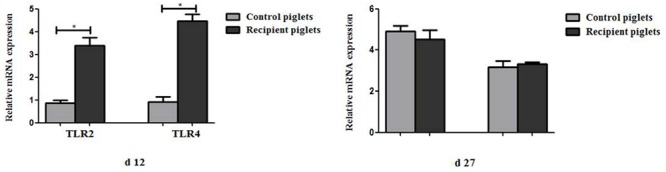
Relative mRNA expressions of TLR2 and TLR4 in colon mucosa of piglets orally treated with or without exogenous fecal microflora. Data are expressed as the mean ± SEM. ^∗^*P* < 0.05.

The relative expression of TLR2 and TLR4 in the colon is shown in **Figure 14**. Compared with control piglets, TLR2 and TLR4 in the colon of recipient piglets were significantly increased at day 12 (*P* < 0.05). TLR2 and TLR4 in the colon of recipient piglets at day 27 were not affected (*P* > 0.05).

## Discussion

The structure of intestinal microbial flora is not only affected by genetic factors, but is also susceptible to antibiotics, diseases, diets, environment as the starting early in life (Schroeder and Bäckhed, 2016). Infancy is a key period of colonization of gut microbial flora, during which microbiota structure is unstable and susceptible to the surrounding environment (Clemente et al., 2012). In this study, DLY newborn piglets were selected to receive fecal microbiota transplantation from healthy adult Jinhua pigs. The results showed that the relative abundance of *Firmicutes* and *Proteobacteria* in the colon of recipient piglets was increased, moreover, *Firmicutes* and *Bacteroidetes* were the two most abundant phyla of these piglets, and this result was similar to the previous study that *Firmicutes* and *Bacteroidetes* had an absolute advantage in mouse gut (Kulecka et al., 2016). In this study, the ratio of *Firmicutes* to *Bacteroidetes* was increased in recipient piglets, and the relative abundance of *Firmicutes, Oscillospira, Prevotella* was also enhanced. *Firmicutes* can maintain intestinal health by producing short-chain fatty acids, inhibit inflammation and provide energy for intestinal epithelial cells (Gophna et al., 2017). *Firmicutes* is also closely related to animal energy metabolism, and it is conducive to animal energy absorption of food under the condition that the proportion of *Firmicutes* is more than *Bacteroides* (Turnbaugh et al., 2006). *Oscillospira* can use host glycans as a source of energy, *Oscillospira* is also probably associated with the production of the short-chain fatty acid butyrate (Konikoff and Gophna, 2016). *Prevotella* is associated with digestion of carbohydrates (Wu et al., 2011). *Veillonellaceae* is associated with dietary intervention and pro-inflammatory (an increased abundance of *Veillonellaceae* was found in IBD, IBS, and cirrhosis patients) (Gevers et al., 2014; Haberman et al., 2014; Shukla et al., 2015; Bonder et al., 2016).

This study also showed that *Escherichia* of recipient piglets was decreased. *Escherichia coli* are a typically pathogenic species that infects intestine, and causes diarrhea and other serious gastrointestinal symptoms, and includes types such as enterotoxigenic and enteropathic *E. coli* (Camerlink et al., 2010). The above results suggested that intervarietal fecal microbiota transplantation increased the proportion of bacteria that benefit the host, and decreased harmful bacteria in the intestine of recipient piglets. Moreover, homology between the intestinal microbial flora of recipient piglets and donor pigs were closer after FMT, though a portion of bacteria in recipient piglets did not change with the corresponding bacteria in the donor. Similar to the structure of microbiota composition in donor pigs, the relative abundance of *Firmicutes, Prevotellaceae, Paraprevotellaceae, Prevotella* of recipient was increased. For these bacteria, receptor piglets showed a donor-like succession, which is consistent with the previous reports (Duca et al., 2014). The results also showed that *Bacteroidaceae, Veillonellaceae*, and *Bacteroides* of the recipient piglets were lower than the control, although it had a higher proportion in Jinhua pigs. Such shift phenomenon has also been reported in other studies (Pang et al., 2007). It might be due to the complex interaction between microbial flora and colonization. Up to now, there were few studies about intervarietal fecal microbiota transplantation induced changes in the structure of intestinal microbial flora in pigs.

As the gut of a newborn pig is not fully developed, intestinal microbial floras are in an unstable state. At the same time, the development of its immune system is not mature enough, thus piglets are susceptible to a variety of pathogens (Xu et al., 1992) Piglets are weak in resistance, diarrhea would happen under this condition. Diarrhea would lead to the decrease of survival rate and growth retardation of piglets. The proportion of *Bifidobacterium* would decrease, *Enterobacterium, Bacteroides* and *Clostridium* would increase when diarrhea happens (Vondruskova et al., 2010). Clinical practice trials showed that the patients with toxic megacolon caused by *C. difficile* infection were treated by FMT successfully, and the frequency of diarrhea was decreased rapidly (Gweon et al., 2015). The diarrhea incidence of recipient piglets was decreased significantly in this study. As mentioned above, *Escherichia* are connect with many diseases including diarrhea (Camerlink et al., 2010). It indicated that the decrease of diarrhea incidence might ascribe to the reduction of colonic *Escherichia*. Intestinal ecosystem of piglets is closely related to its growth characteristics (Ramayo-Caldas et al., 2016). The microbial structures of the heavier body weight piglets are different from the lighter ones (Han et al., 2016). The increase of bacteria mentioned above, which associate with nutrient absorption and metabolism might also lead to the weight gain of the recipient. Consistently, the piglets inoculated with fecal microbiota suspension performed better than the control piglets in this study, and the differences in the intestinal microbial composition between recipient and donor led to the differences in their weight gain. Moreover, the decrease of diarrhea incidence might also contribute to weight gains of recipient piglets.

The development of intestine in newborn piglets is rapid, which include tissue growth, morphology changes and functions maturity. The villi capillaries of conventional mice were better developed compared to germfree mice, which suggested that the gut microbiota contribution to the development of the intestinal villi (Hooper, 2004). The growth rate of crypts affects growth and atrophy of the intestinal villi, the reduction of crypt depth indicate an increase of epithelial cell maturation rate and absorptive capacity. So the decrease of crypt depth in recipient piglets in this study indicated the enhanced ability of absorption and digestion in the small intestinal epithelium. As shown under microscope, jejunal villi of recipient piglets were smooth with luminal integrity, while the control group showed broken luminal integrity. The scanning electron microscopes result indicated that that intestinal villus morphology was improved by fecal microbiota suspension.

The integrity of the intestinal barrier structure is the basis for maintaining normal intestinal function. Tight junction is an important part of intestinal mucosa mechanical barrier function, which prevents bacteria and other antigens from spreading over the epithelial cells (Ulluwishewa et al., 2011). Tight junction proteins are often used as an indicator for the function of the barrier and the permeability in the intestine, including: occluding, ZO-1, and cladding. Improvements of intestinal barrier integrity were associated with tight junction proteins expression levels (Ulluwishewa et al., 2011). Microbiota can lead to the increase of tight junction proteins at cell boundaries, and prevent or reverse the harmful effect of pathogens in the intestine in some cases (Ulluwishewa et al., 2011). Studies have shown that probiotics can regulate tight junctions through bacterial metabolites (Hsieh et al., 2015). The expressions of tight junction proteins in the intestine of recipients were increased in this study.

As the first line of defense against intestinal microbial invasion host, goblet cells play a key role in the identification of intestinal microorganism (Shen, 2009). Intestinal microbes can directly regulate the goblet cell function by delivering local biologically active factors. And the functions of goblet cells can also be changed by host-derived bioactive factors which are produced by active epithelial or underlying lamina propria cells after contacting with intestinal bacteria (Deplancke and Gaskins, 2001). Intestinal goblet cells in the sterile-fed mice were fewer in number and smaller in volume as compared with that of the conventionally fed mice, which demonstrated that microbe regulates the number of goblet cells (Deplancke and Gaskins, 2001). In the present study, the number of goblet cells in the colon was higher than in jejunum both on days 12 and 27 which was consistent with the Karam (1999) report. From duodenum to the ileum, and jejunum, the ratio of goblet cells in all epithelial cells occupied 4, 6, and 12%. In the distal colon, the goblet cells proportion increased to 16%. This coincides with a gradual increase in the number of microbes from the proximal intestine to the colon. Goblet cells mainly secrete making that cover intestinal epithelial surface, and play the role of mechanical protective barrier (Linden et al., 2008). Mucins lubricate intestine and protect it from the damage of potential pathogens, mutagens, as well as mechanical and chemical damage (Neutra and Forstner, 1987). The predominant gel-forming mucin in the intestine is MUC2, which could be expressed by goblet cells throughout intestine (Johansson and Hansson, 2016). Without microbiota in the intestinal tract, the thickness of the mucous layer will become thinner, and bacteria can contact with the epithelium directly, translocate and trigger the submucosal immune system to cause an immune response (van Vliet et al., 2010; Johansson and Hansson, 2016). Different bacteria species in the colon of mouse also led to the differences in mucin production capacity (Caballero et al., 2015). So in this study, the different structure of intestinal microbial flora may also cause the different expressions of MUC2 in the intestine. The intestinal microbiota induced mucin production and antibiotic administration resulted in thinning of the mucus barrier, thereby increasing susceptibility to bacterial invasion (Wlodarska et al., 2011). The present study showed that mRNA and protein expressions of MUC2 were up-regulated in recipient piglets. The increase of MUC2 might be attributed to the increase of the number of goblet cells. The results indicated that the exogenous fecal microbiota suspension had a beneficial effect on the development of intestinal mucous barrier in recipient piglets. So in this study, intestinal morphology and intestinal barrier function was improved via intervarietal fecal microbiota transplantation.

There are a wide variety of antimicrobial peptides, which aid congenital barrier resist microbial infection. As the first line of innate immunity in mammals, antibacterial peptides are a class of small molecules that are widely found in animals. The expression of β-defensin 2 gene is inducible and induced by intestinal microbes in the intestine of pigs (Veldhuizen et al., 2006). The immune system of newborn piglets is imperfect, antimicrobial peptides are the key effector molecules of innate immunity at this stage. The expression of antimicrobial peptide gene in the intestine of mice increased gradually with age after birth (Ménard et al., 2008). β-defensin 2 increased gradually with age was also found in this study, the expressions of β-defensin 2 in ileum of recipient piglets on day 27 were higher than that of piglets on day 12. Moreover, the expressions of β-defensin 2 in recipient piglets were enhanced. The expressions of defensin vary in breeds of piglets. It has been reported that the expression level of defensin gene in the intestine of Jinhua pig was higher than that in Landrace pig (An et al., 2011). And they consider that β-defensin play the key role during each stage of piglets growth and development that the expression of β-defensin is different in different breeds might contribute to the various disease resistance of pigs. We found that the expressions of β-defensin 2 in recipient piglets were higher than that of the control in this study. It indicated that intervarietal fecal microbiota promoted expressions of intestinal antimicrobial peptides in DLY recipient piglets. Increased expressions of antimicrobial peptide contributed to the improvement of the resistance of recipients to diseases.

As predominant immunoglobulin isotype on most mucosal surfaces (Otten and van Egmond, 2004; Snoeck et al., 2006), secretory immunoglobulin A (sIgA) can protect intestinal tract from dietary and microbial antigens (Bakker-Zierikzee et al., 2006). Except for the inhibition of adherence and invasion of potentially harmful antigens into mucosa, sIgA also can neutralize toxins and virulence factors from microbial pathogens (Brandtzaeg, 2003). The innate inflammatory response of neonates is not mature enough to prevent pathogen invasion (Walker, 2010). The relationship between microbes and sIgA is mutual adjustment. SIgA in the intestinal mucosa of mice that the intestinal microbial flora imbalanced caused by antibiotic were increased after receiving fecal microbiota suspension (Li et al., 2015). The balance of original intestinal microbiota in recipient piglets would be disrupted after fecal suspension was treated and the optical density of sIgA^+^ cells in the colon was also increased in recipient piglets consistently. More and more bacterial colonization facilitates the maturation of mucosal immune system and is associated with the production of the large amount of sIgA, which stabilizes the microbiota-host interaction (Pabst et al., 2016). Exogenous fecal microbiota suspension stimulated proliferation of sIgA^+^ cells and the development of the intestinal immune system of piglets.

The composition of intestinal microbiota and function of the immune system is closely related (Round and Mazmanian, 2009). As a sensor for microbial infection, TLRs are critical for initiation of inflammation and immune defense responses (Rakoff-Nahoum et al., 2004). TLR recognized bacterial ligands that are not only unique to pathogens, but shared by all bacteria, and also produced by symbiotic microorganisms (Chen et al., 2010). Previous studies have shown that intestinal microbiota can activate TLR2/TLR4 on the luminal surface of epithelial cells and may subsequently improve intestinal barrier function through promoting the assembly of intestinal tight junction-associated molecules as well as regulating the proliferation and apoptosis of epithelial cells (Rakoff-Nahoum et al., 2004; Fukata et al., 2006; Cario et al., 2007). Stimulated by exogenous fecal bacteria, TLR2 and TLR4 of recipient piglets on day 12 were increased. As mentioned above, exogenous microbial flora might break the original balance of microbiota, therefore, TLRs in the intestine of piglets were significantly increased by the stimulation of exogenous fecal microbiota. However, there were no significant differences in the expressions of TLRs on day 27, which might indicate that intestinal microbiota of recipient piglets was reestablished and the new homeostasis stablished. The activation of TLRs by commensal bacteria is essential for maintaining homeostasis in the colon (Ben-Neriah and Schmidt-Supprian, 2007). Sensing of commensal bacteria by TLRs in epithelial does not elicit an inflammatory cascade but is required for the maintenance of the epithelial barrier and contributes to TLR-dependent intestinal homeostasis observed *in vivo* (Rakoff-Nahoum et al., 2004; Cario and Podolsky, 2005). Moreover, the induced TLR receptors in intestinal epithelium also can stimulate an innate immune response and activate the PP to secrete antimicrobial peptides (Wang et al., 2016), and lead to the induction of MUC2 (Ikeda et al., 2007). The study indicated that the expression of TLRs were up-regulated under the condition that intestinal microbial flora was imbalanced and didn’t change when the structure of intestinal microbiota restored to steady state.

In summary, some characteristics of donor pigs could be transferred to recipient neonatal piglets by fecal microbiota suspension in this study. Exogenous fecal microbiota suspension changed the structure of intestinal microbial flora as well as contributed to the improvement of intestinal morphology, the development of the intestinal mucosal barrier and innate immunity in recipient piglets. Therefore, the recipients’ resistance to disease was enhanced, diarrhea was reduced and weight gain was raised. Fecal microbiota transplantation might be an efficient way to improve young animals’ resistance to disease by changing the structure of intestinal microbial flora.

## Author Contributions

XH conceived and designed the experiments. LH and SG performed the experiments, analyzed the data and drafted the manuscript. YL, SC, XF, and XY performed the experiments and analyzed the data.

## Conflict of Interest Statement

The authors declare that the research was conducted in the absence of any commercial or financial relationships that could be construed as a potential conflict of interest.

## Footnotes

1 http://ccb.jhu.edu/software/FLASH/

2 http://qiime.org/scripts/split_libraries_fastq.html
